# Supplementary material for: Resurrecting ancestral genes in bacteria to interpret ancient biosignatures
Source: Philos Trans A Math Phys Eng Sci. 2017 Nov 13;375(2109):20160352. doi: 10.1098/rsta.2016.0352 (PMC5686408; doi:10.1098/rsta.2016.0352)
Supplement: Supplementary Information [file rsta20160352supp1.pdf]

Supplementary Figures

Supplementary Figure 1: Maximum-likelihood phylogenetic reconstruction of the carbonic anhydrase (CA). We applied the same analysis as shown for Figure 2, but no clade has been collapsed, and all bootstrap values are shown. Leaves are colored according to their taxonomic classification: red, Proteobacteria; blue, Cyanobacteria; green, Archaea; orange, Actinobacteria; purple, Firmicutes. When available, the organism name is prefixed with the phylum and class the organism belongs to.

Supplementary Figure 2: Maximum-likelihood phylogeny of carbonic anhydrase homologs present in representative of each genus of the Cyanobacteria and a few outgroup species. The tree was inferred with RAxML. Bootstrap support is shown for each branch. Shades of blue represent different families of Cyanobacteria. The scale represent the number of substitutions per site.

Supplementary Tables

Supplementary Table 1: List of 388 genomes initially searched for the presence of CA. The file is a partial output of phyloSkeleton.

Supplementary Table 2: List of 79 cyanobacterial genomes and 4 outgroups. The file is a partial output of phyloSkeleton.
