# Supplementary figures and images for: Resurrecting ancestral genes in bacteria to interpret ancient biosignatures
Source: Philos Trans A Math Phys Eng Sci. 2017 Nov 13;375(2109):20160352. doi: 10.1098/rsta.2016.0352 (PMC5686408; doi:10.1098/rsta.2016.0352)

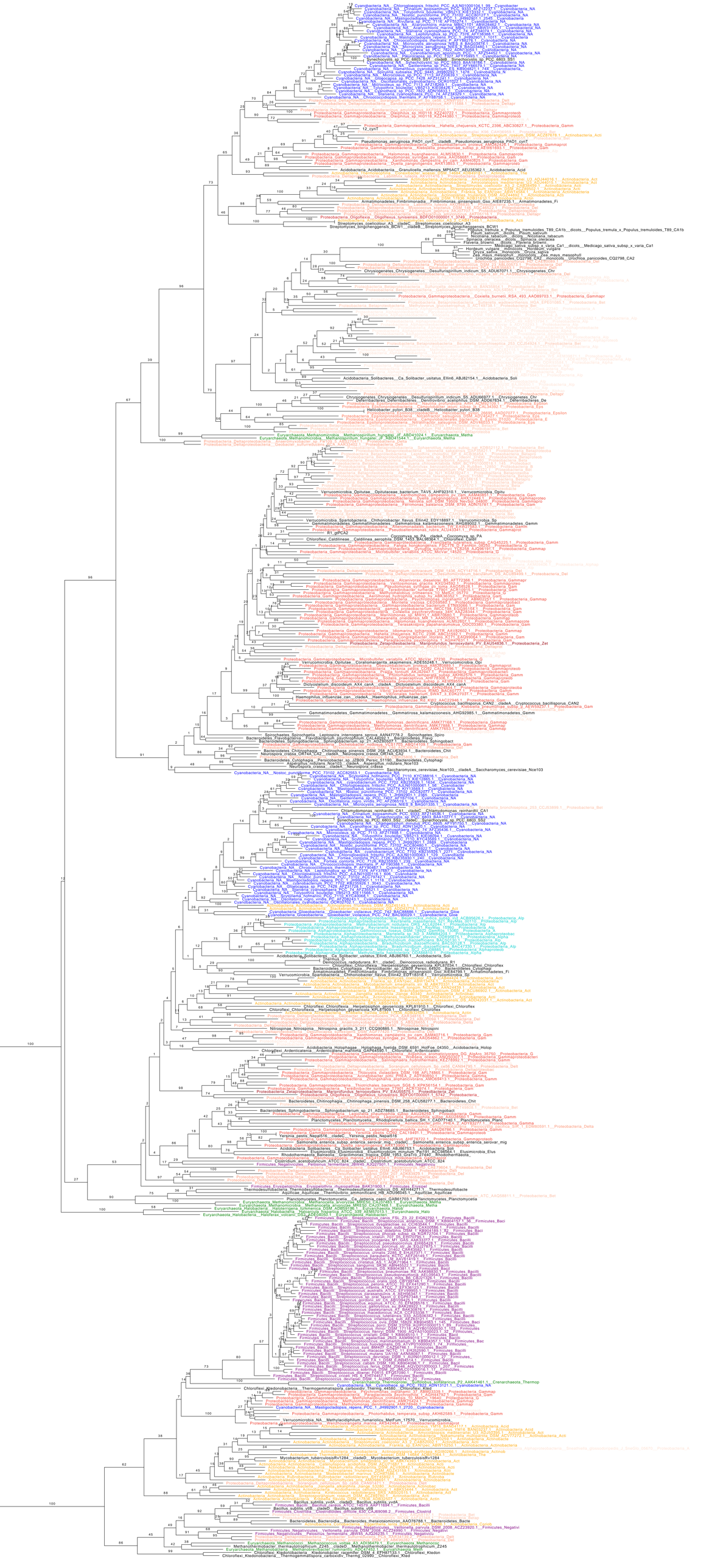

Supplement: Figure S1 [file rsta20160352supp2.pdf]

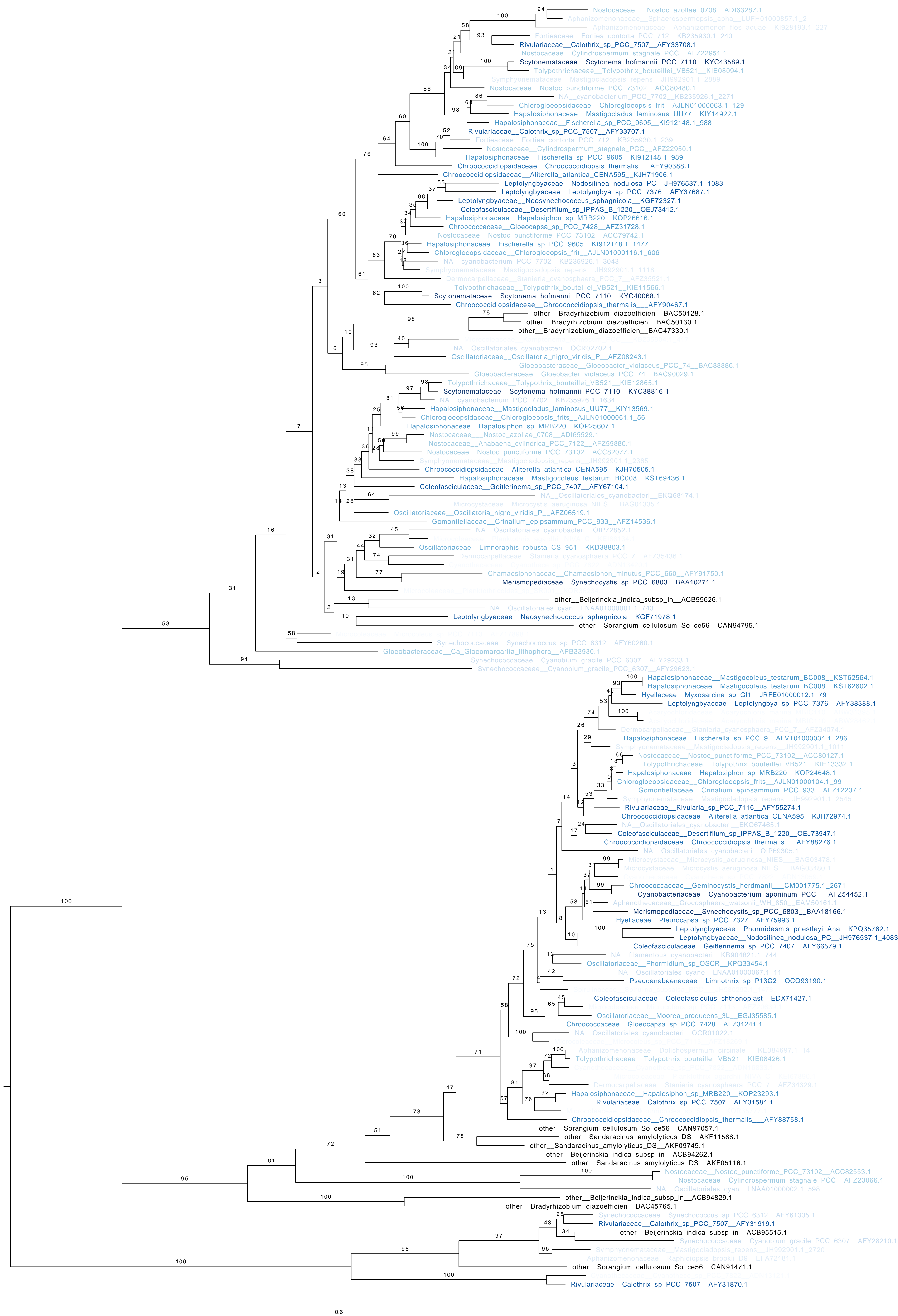

Supplement: Figure S2 [file rsta20160352supp3.pdf]
